# Hepatitis C: milestones from discovery to clinical cure

**DOI:** 10.1186/s40779-020-00288-y

**Published:** 2020-12-01

**Authors:** Wei Hu, Chao Zhang, Ji-Jing Shi, Ji-Yuan Zhang, Fu-Sheng Wang

**Affiliations:** 1 Medical School of Chinese PLA, Beijing, 100842 China; 2 Department of Infectious Diseases, the Fifth Medical Centre of Chinese PLA General Hospital, National Clinical Research Centre for Infectious Diseases, Beijing, 100039 China; 3 The Central Laboratory, the First People’s Hospital of Zhengzhou, Zhengzhou, 450004 China

## Abstract

On October 5th, 2020, Drs. Harvey J. Alter, Michael Houghton and Charles M. Rice were rewarded with Nobel Prize in Physiology or Medicine for “the discovery of hepatitis C virus (HCV)”. During the past 50 years, remarkable achievements have been made in treatment of HCV infection: it has changed from being a life-threatening chronic disease to being curable. In this commentary, we briefly summarized the milestone events in the “scientific journey” from the first report of non-A, non-B hepatitis and discovery of the pathogen (HCV) to final identification of efficacious direct-acting antivirals. Further, we address the challenges and unmet issues in this field.

## Background

The 2020 Nobel Prize in Physiology or Medicine was awarded to Drs. Harvey J. Alter, Michael Houghton and Charles M. Rice for the discovery of hepatitis C virus (HCV). HCV is a single-stranded, positive-sense RNA hepatophilic virus. In contrast to oral transmission of hepatitis A virus (HAV) and bodily fluids-mediated transmission of hepatitis B virus (HBV), HCV is primarily transmitted through blood transfusion and intravenous drug use. After acute HCV infection, approximately 75–85% of the patients develop chronic hepatitis C (CHC). Patients with CHC are prone to hepatic cirrhosis, chronic liver failure and hepatocellular carcinoma [1]. HCV infects more than 170 million people worldwide, and approximately 10 million in China [1, 2], and thus represents a huge healthcare and economic burden.

## Discovery of HCV

**Fig. 1:**
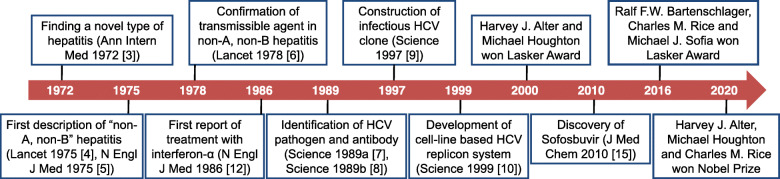
Milestones in the discovery of HCV and development of treatment

Discovery of HCV began with the finding a novel type of hepatitis in patients who received blood transfusion [3] (Fig. 1). HAV and HBV were not present in such patients [4, 5]. Alter and colleagues coined the term “non-A, non-B hepatitis” in 1975 [4]. They collected plasma/serum samples from a blood donor with chronic hepatitis and 4 people who developed “non-A, non-B hepatitis” after receiving blood transfusion, and injected these samples to 5 chimpanzees. As they suspected, all 5 chimpanzees developed hepatitis, as evidenced by elevated alanine aminotransferase as well as liver pathological changes, confirming the presence of a yet unknown transmissible agent in the blood of patients with non-A, non-B hepatitis [6].

In 1989, Houghton and colleagues constructed a random-primed complementary DNA library using plasma samples from patients with non-A, non-B hepatitis [7]. One clone in this library was not derived from host DNA, and appeared to be from a novel RNA virus belonging to the *Flavivirus* family (at least 10,000 nucleotides and is positive-stranded) [7]. Houghton and colleagues named this novel virus as HCV and reported a diagnostic assay using yeast-expressed recombinant polypeptide based on HCV genome to capture viral antibodies in the same journal that published the sequencing information [8].

Rice and colleagues later constructed a full-length clone of HCV complementary DNA that could transcribe an infectious RNA variant of HCV [9]. Upon intrahepatic inoculation of this clone, chimpanzees developed chronic hepatitis, with production of antibodies against HCV and viral replication in the blood [9]. Subsequently, Bartenschlager and colleagues developed an in vitro cell culture using a human hepatoma cell line to replicate HCV [10]. This cell-based model is indispensable in revealing the biological features of HCV as well as developing anti-HCV agents.

## Identification and application of DAAs

Treatment of CHC with interferon-α, alone or in combination with ribavirin, is effective, but only in approximately 30% of the patients [11, 12]. Direct-acting antiviral (DAA) medications that directly block the activity of HCV non-structural proteins revolutionized the treatment of CHC [13]. PSI-6130 was the first DAA that inhibits the HCV NS5B RNA polymerase and selectively suppresses HCV replication [14], but clinical development effort was abandoned due to low bioavailability. PSI-7851, also known as sofosbuvir, is a PSI-6130 derivative [15], and improved in vivo efficacy against HCV of various genotypes [16, 17]. Ledipasvir is a DAA that inhibits the HCV NS5A RNA polymerase [18, 19]. The combination of sofosbuvir and ledipasvir achieved >95% response rate with minimal side effects in adult CHC patients [13]. The latest development is Vosevi. As a polypill (sofosbuvir, velpatasvir and voxilaprevir in a single tablet), Vosevi is easy to use, and has many advantages, including improved pharmacokinetics and strong antiviral activity. There has been speculation that with Vosevi and other treatments in the pipeline, we are now approaching the ultimate WHO goal of eradicating HCV by 2030.

## Conclusion and perspective

Despite of the optimism brought about by the advances in the past decades, many challenges are ahead [20]. First, there is no effective vaccine in sight. Also, cured patients often fail to gain fully reinvigorated immunity. Second, more efforts must be made to identify and treat asymptomatic patients. Third, high risk behaviors (such as needle sharing) must be curbed. Last but not least, effective treatments must be made accessible to all infected people.

## Data Availability

Not applicable.

## Abbreviations

CHC: Chronic hepatitis C
HAV: Hepatitis A virus
HBV: Hepatitis B virus
HCV: Hepatitis C virus
